# A Study on the Soundscape of Underground Commercial Space in Lu’an City and Hefei City, China

**DOI:** 10.3390/ijerph20031971

**Published:** 2023-01-20

**Authors:** Su Wang, Huaidong He, Fulong Li, Qingqing Xiao

**Affiliations:** 1 College of Art, Anhui University, Hefei 230601, China; 2 School of Environment and Tourism, West Anhui University, Lu’an 237012, China; 3 Food and Environment, School of Biology, Hefei University, Hefei 230601, China

**Keywords:** soundscape, underground space, above-ground space, background music, public health

## Abstract

Soundscape is an important part and one of the main factors of the underground space environment. Field surveys were conducted to evaluate the soundscape of underground commercial spaces and to compare it with the soundscape of the above-ground commercial spaces between two cities (Lu’an City and Hefei City) in China, consequently presenting the construction strategy of the soundscape of underground commercial spaces in urban areas. The results showed that the sound in the shopping center, which people found comfortable, was at the lower to intermediate level. The main sounds that people perceived as “general” sounds were environmental sounds such as music, the humming of the air conditioning, people talking, walking, and the hawking of the stores. Nevertheless, “very comfortable” sounds were background music and the sound of live performances, which were indicated in the majority of people’s opinions on evaluating a comfortable feeling, thus reflecting the impact of the sound of mall music on people’s cognitive psychology. Therefore, it is necessary to control the volume of environmental noise at a certain level so that people’s health is not adversely affected. It also helps shoppers to feel more comfortable psychologically and physiologically.

## 1. Introduction

With the development of urbanization and the increase in population density, the “urban syndrome”, represented by land shortages, traffic congestion, and environmental pollution, has become increasingly severe. Potentially, developing and utilizing urban underground spaces can alleviate the problems mentioned above [1,2,3]. Urban underground spaces are underground public places for urban residents, such as underground shopping streets, stations, tunnels, corridors, and commercial and transportation facilities, which are always crowded [4]. Compared with above-ground space, the underground space is relatively closed off from the natural environment and is less affected by it; it has also relatively poor physical conditions. For example, the sound pressure level of the same sound source in an underground space environment is generally higher than that in the above-ground space due to the closed nature of the underground space and spatial characteristics such as its shape [5]. It may be difficult to solve the acoustic problem of underground space by applying a traditional acoustical theory, which makes it difficult to create the desired acoustic environment of the underground space [5,6,7,8,9]. Therefore, the issue of how to effectively solve the noise problem of underground space, given its ability to improve the quality of the acoustic environment of urban space, has recently become a concern. This research focuses on investigating the relationship between underground space and the acoustic environment, thus contributing to the development of acoustics.

The term “soundscape” was first proposed by Canadian musician R. Murray Schafer in the 1970s [10]. The soundscape is a combination of natural, artificial, historical, and cultural sounds, as well as the sounds of human activity. It reveals the relationship between human hearing, the sound environment, and society, and regards the sound environment as a resource rather than a physical parameter [9]. Soundscape research involves many disciplines, such as acoustics, musicology, design, psychology, and architecture. As far as the evaluation and creation of a soundscape are concerned, the sounds existing in nature and melodies of physical motion can cause people to have certain auditory associations. This analog approach can trigger a series of image associations, called “similar thoughts” or “synesthesia” in psychology. Southworth [3] conducted in-depth research on the pleasant and recognizable sounds that evoke “similar thoughts” in the soundscape. The conclusion that can guide the evaluation and construction of soundscape was drawn. For example, a pleasant soundscape should include sound elements with novel and inclusive information to allow people to experience a sense of cultural identity. Soundscape construction relies on its association with the cultural environment. The frequency and intensity of sound in soundscape construction should be maintained at low and medium values [10,11,12,13,14,15]. Southworth’s research has led designers to think about the “similarity” of space and its relationship with space quality. Soundscape construction is considered a new trend of thought that stimulates the potential of designers [15,16,17,18] and challenges the traditional dominance of vision in today’s space design. Previous studies have conducted soundscape change [19,20,21,22], quality assessment [23,24], spatial correlation [25], and restoration research [26] for different spatial environments. In terms of sound scene preferences, the difference in sound source types [27], sound source preferences, and perception experiences [28] have been the focus of attention in previous research. However, these studies mainly focused on urban forests, green spaces, and communities, and paid little attention to underground spaces.

The soundscape is an important part of the underground space environment. The impact that urban underground spaces have on the physiological and psychological health of people, and the ways to create a good soundscape in underground spaces are the issues that people generally care about and need to be studied. For example, it is easy to make people uncomfortably anxious in a closed environment [29]. The most typical example of an urban underground space is a commercial space that can potentially supplement the residential living space. At present, there are several problems related to the lack of balance between the underground commercial space, the supporting facilities of the above-ground commercial space, and the lack of systematic development and utilization of the underground commercial space [30]. Kang [4] studied the acoustic environment of Harbin’s underground space. However, the basic acoustic data about these underground spaces are very limited. Therefore, this work aims to study the soundscape of underground commercial spaces by analyzing previous case studies to deepen the evaluation and understanding of specific spaces from the auditory perspective and also proposes a new strategy for creating a soundscape in the underground space.

Underground commercial spaces are normally divided into two types: the street type and the square type, depending on the different shapes of the spaces. The street type of underground commercial space is usually independent, while the square type of underground space tends to be attached to the above-ground commercial building as a basement [31,32,33,34,35,36]. In this study, the underground commercial street in Jiefang Road (street type), Lu’an City, Anhui Province, and the underground commercial street Zhixin Cheng (square type) in the heart of Hefei City were selected as the research subjects for analysis. This study aims to reveal the basic acoustic characteristics of the underground space and the sound preference response of the people in the region.

## 2. Methods

### 2.1. Study Design

Soundscape surveys mainly involve the methods of subjective surveys and objective measurement. A questionnaire survey was used as a subjective investigation to collect the subjective feelings of respondents, and the objective measurement was to determine the sound pressure level (SPL) and other environmental indicators. The subjective and objective methods determined the accuracy of the survey results together. Although soundscape evaluation is complex, it can still be accomplished by studying the relationships between sound, listener, space, and environment [37,38,39].

**Figure 1:**
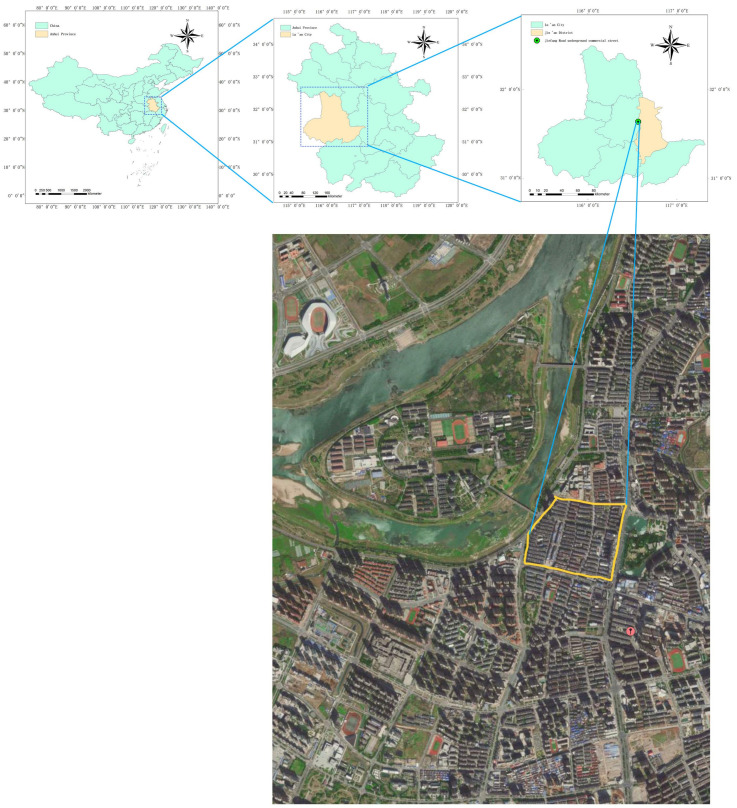
Study area and sampling sites in Lu’an City.

**Figure 2:**
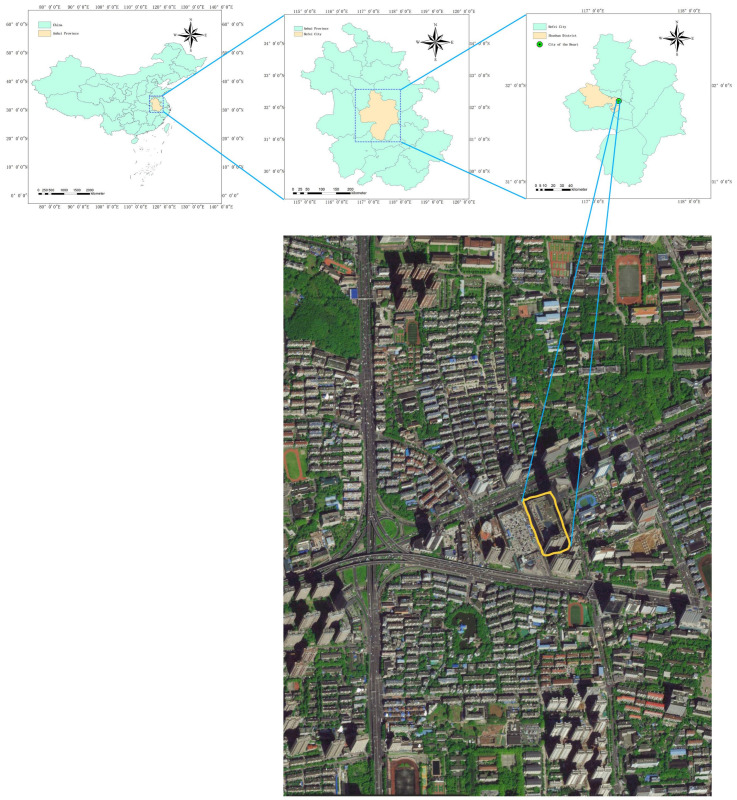
Study area and sampling sites in Hefei City.

The soundscape of the underground commercial spaces of Jiefang Road in Lu’an City (Figure 1) and Zhixin Cheng in Hefei City (Figure 2) was evaluated via subjective and objective methods. To reveal the basic acoustic characteristics of the underground spaces and people’s sound preference in the area, the sound pressure level (SPL) was systematically measured to express the objective property after the identification of sound source types, and the subjective questionnaire survey of the sound preference was conducted via Likert scale in the typical underground spaces selected [16]. Before the questionnaire survey, the respondents confirmed whether they were willing to accept the inquiry and that their hearing is normal. The research focused on the survey of sound sources and the sound preference evaluation in the underground commercial space of Jiefang Road in Lu’an City, while the comparative analysis of the subjective evaluation was the main focus on the sound and scenery of the above-ground and underground commercial spaces of Zhixin Cheng in Hefei City.

### 2.2. Jiefang Road in Lu’an City—Investigation and Evaluation of Sound Source in Underground Commercial Spaces

#### 2.2.1. Overview of the Study Area

**Figure 3:**
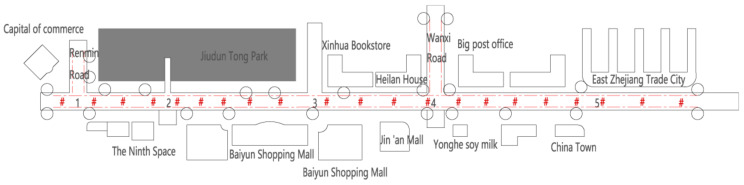
Layout of the underground commercial street and sound source survey in Jiefang Road, Lu’an (1–5: sampling points).

**Figure 4:**
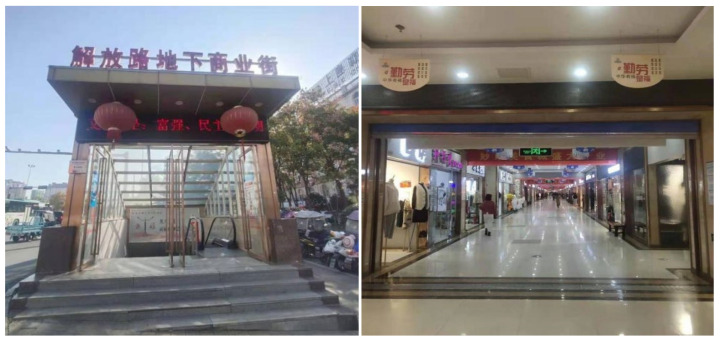
Pictures of the underground commercial street on Jiefang Road in Lu’an City.

The sound source analysis is one of the most important parts of soundscape research. Its purpose is to summarize the types of sound sources through field investigation and the identification of dominant sound sources in the environment and to determine the construction countermeasures. The underground commercial street selected is located on Jiefang Road in Lu’an City and has been in operation since 2014 after renovation. It is about 835 m long and 21 m wide, with a total area of about 17,535 square meters. The main reasons for choosing this business district as the research subject are as follows: This commercial street is located in the downtown area of Lu’an City and is a large underground business center built as a joint construction project of civil air defense. Its spatial form is a single type, i.e., a typical street commercial space in a shape of a rectangle (Figure 3). There are a large number of customers of different ages and needs in this business place every day, which guarantees the quantity and diversity of samples (Figure 4).

#### 2.2.2. Questionnaire

Before the implementation of the questionnaire, the types of sound sources in the underground space of Jiefang Road in Lu’an City were evaluated through the field survey. The sound source types included the sound of mall music, the sound of live performance music, the humming of the air conditioning, people talking, walking, and the hawking of the stores, etc., which could affect respondents’ sound preference.

**Figure 5:**
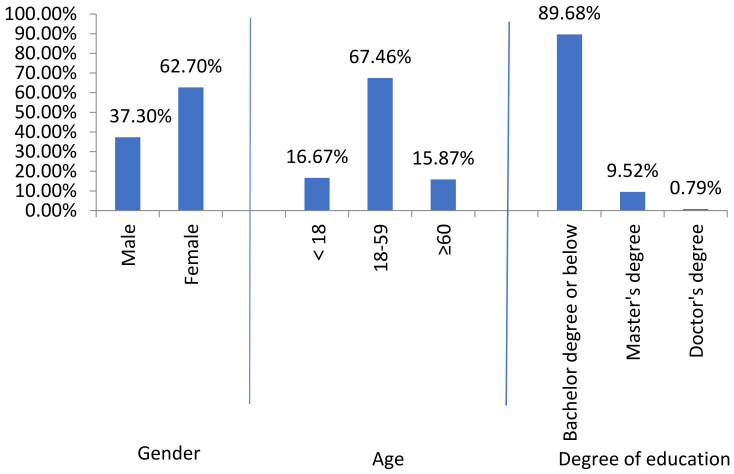
Basic composition of interviewees in underground commercial space on Jiefang Road in Lu’an City.

For the convenience of investigation, the questionnaire was mainly conducted through the interviewees’ dictation and the recorder’s filling. The questionnaire consisted of two parts: collecting the background information of the respondents and evaluating their perception of the sound landscape (the main part of the questionnaire). The purpose of the first part was to obtain the basic demographic characteristics of the respondents, such as gender, age (grouped into under 18, 18–59, and over 60), and education level (bachelor’s degree or below, master’s degree, and doctor’s degree) (Figure 5). The second part of the questionnaire was the evaluation of the respondents’ preference concerning the sound landscape, as shown in the sound scale (Table 1). Along with the ISO regulation of the soundscape preference measurement, the Likert scale and a total of four subjective questions were considered to measure the type of sound source perceived by people. The answer to each question was divided into 5 levels and was recorded as 1–5 points, respectively. The total score of each response was the sum of the scores in all the questions, which indicated the strength of the respondent’s attitude or different states on this scale.

#### 2.2.3. Survey Methodology

According to the spatial characteristics of the commercial street, 128 questionnaires were collected at 5 points (Figure 3), and 126 of them were valid. The survey targets on the site were customers and merchants in the underground commercial street. In order to obtain accurate results, the survey was conducted on a Sunday when there was a large flow of people, using the method of equidistant sampling. Regarding the survey of merchants, 1 household was randomly selected from every 10 households according to the order of stalls. For the customer’s choice, one customer was selected every 10 min from 9:00 to 16:00 due to the high mobility of people.

Along with the questionnaire survey, the SPL was measured using the Norsonic121 and BSWA801 sound level meters and then analyzed using 01 dB software (01 dB Stell). During the measurement, the temperature was 25 °C, and the relative humidity of the tested area was about 65%. The measurements were made according to the ISO sound level standards prepared by International Organization for Standardization (ISO 2003, Acoustics—description, measurement, and assessment of environmental noise. Part 1: Basic quantities and assessment procedures).

### 2.3. Zhixin Cheng in Hefei City—A Comparative Analysis of Subjective Evaluation of Soundscape in Above-Ground and Underground Commercial Space

#### 2.3.1. Overview of the Study Area

The soundscape of the above-ground space is affected by the surrounding environment, as well as its own sound. By contrast, the soundscape of the underground space is less affected by the above-ground environment. In order to further understand the soundscape of the underground space, a comparative study of the soundscapes of above-ground and underground spaces was conducted from the perspective of space users, considering their feelings and evaluating the sound environment (sound evaluation and satisfaction evaluation of acoustic environment) [37,38,39,40].

**Figure 6:**
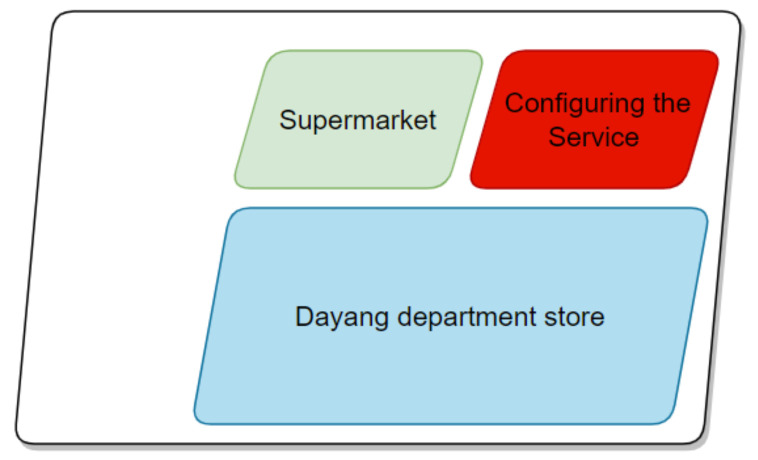
Layout of the underground floor of the Zhixin Cheng of Hefei City.

**Figure 7:**
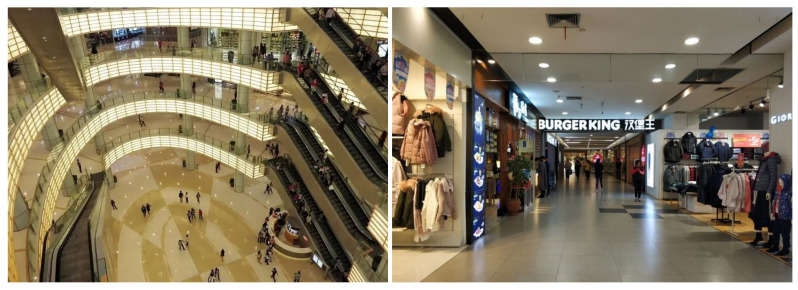
Pictures of Zhixin Cheng, Hefei City.

Regarding both above-ground and underground commercial spaces, the understanding of the soundscape should initially focus on the business circle and the commercial space, followed by spatial positioning and location. The business district is divided into main, secondary, and marginal business districts. The main business district refers to the geographical area where about 70% of the customers are located. The secondary business district refers to the area where the main business district extends outward, and about 20% of the customers are in this area. The marginal business district is the area where the remaining 10% of the customers visit [41,42,43,44,45,46]. In this study, we selected Zhixin Cheng (square type), which is located in the core area of Sanli’an main business district and the hub of urban trunk roads in ShuShan District, Hefei City, China. It is a large-scale comprehensive shopping center with a total floor area of about 350,000 square meters, which contains apartments, office buildings, and shopping centers. The shopping center covers an area of 120,000 square meters, from the first floor underground (Figure 6) to the seventh floor above the ground (Figure 7). The first floor underground is a typical square type of underground space.

#### 2.3.2. Questionnaire

In this study, the first floor underground, as well as the first, third, and fourth floors above the ground were selected as the research areas. The first floor underground and the third floor were selected as the representatives of the shopping centers and commercial complexes, with 202 and 112 people, respectively. The survey was collected from 260 people on the first floor underground and the first floor in the commercial street and was collected from 308 people in the shopping plaza located on the first floor underground and the fourth floor above the ground (Table 2). This part of the study was mainly to investigate consumers’ perception of the comfort of sound sources in Zhixin Cheng, Hefei City.

In addition, the evaluation of the difference between the underground and aboveground space of Zhixin Cheng mainly focused on whether customers can rest after shopping. Because of the above-ground space, customers were mainly shopping, and there was almost no rest space. Therefore, the subjective acoustic comfort of customers who had been resting on their seats in the underground mall (Group A) and those who had not yet sat down to rest in the above-ground mall (Group B) were also compared. There were 50 samples in Group A and Group B, respectively.

### 2.4. Validity Analysis

The reliability of the questionnaire used in this study was tested using Cronbach’s α, which was widely used in social surveys for the calculation of reliability using the reliability coefficient (α = (K/(K − 1)) × (1 − (∑ S_i_^2^)/S_T_^2^)) [10] (S_i_^2^ represents the variance of the Kth test item, and S_T_^2^ represents the sum of the variances of each measurement item). For accuracy (validity), a KMO (Kaiser–Meyer–Olkin) test [11] was conducted, which is widely used for impact factor analysis to determine the accuracy. The coefficients of the KMO ranged from zero to 1, and a higher coefficient indicated better validity. Cronbach’s α and KMO were performed in SPSS 26.0. Finally, the reliability coefficient was obtained, and the validity (accuracy) coefficients were 0.941 and 0.755 (Table 3), respectively, indicating that the reliability and validity of the questionnaire met the requirements.

## 3. Results

### 3.1. Investigation and Evaluation of Sound Source in Lu’an City’s Underground Commercial Space

**Figure 8:**
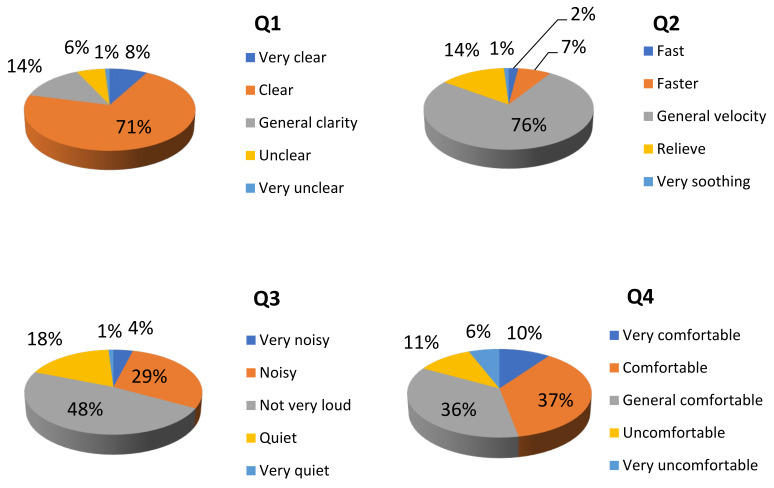
Sound scale of the underground commercial space on Jiefang Road in Lu’an City (*n* = 126, Q1–Q4 are the questions in Table 1. Q1: Subjective evaluation of sound clarity; Q2: Comments on the speed of background music; Q3: Subjective loudness evaluation of sound; and Q4: Evaluation of subjective acoustic comfort).

In this study, the SPL in the commercial street was 55–78 dB. The background music in the mall played popular songs with 70–120 beats per minute and 65 dB. A total of 98.1% of the respondents completed the questionnaire within 10 min, indicating that the questionnaire could be completed within the endurance of most people. The results of Q1 to Q4 (Table 1) in the sound scale are shown in Figure 8. The perception of 71% of the respondents on sound clarity was “clear”, and the perception of 76% of the respondents on the speed of the background music was “general”. The dominant result on the subjective loudness of the sound was “not very loud” (48%), followed by “loud” (29%). Most respondents considered the subjective acoustic comfort as “comfortable” (37%) or “generally comfortable” (36%).

**Figure 9:**
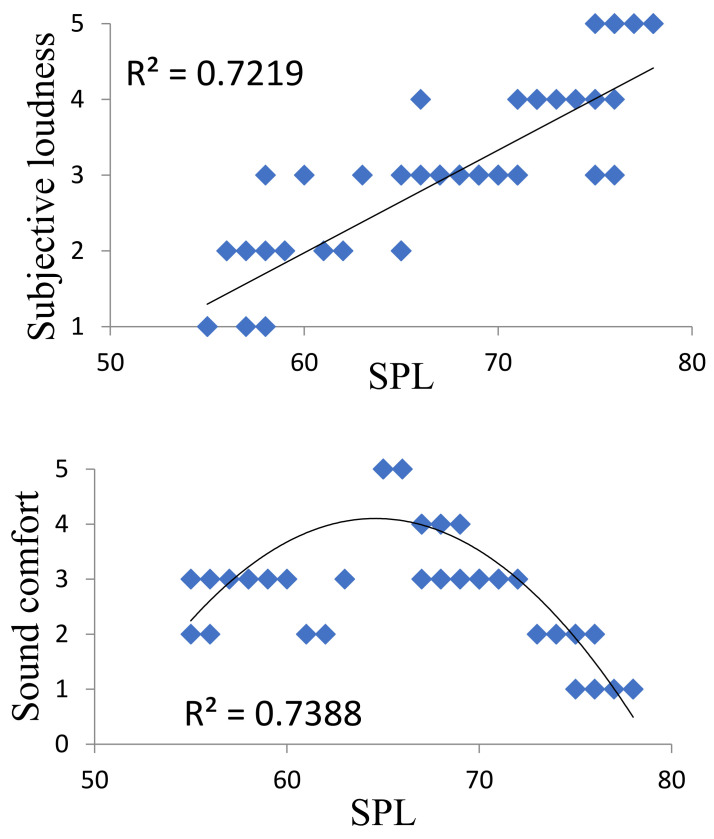
Correlation between subjective loudness, sound comfort, and SPL.

The questionnaire survey, combined with the statistical analysis, showed that the general evaluation of the sound of the underground commercial street was “comfortable” and “general” (*p* < 0.05). In the underground commercial street, the sound comfort changed in the form of a parabola with the increase in the sound pressure level. The subjective comfort was low when the sound pressure level was relatively low and relatively high, but when the environmental sound pressure level was about 65 dB, and the background music speed was 70–80 beats per minute, the customers had a high degree of comfort in terms of environmental sound (Figure 9). The differences in sound comfort between different genders and age groups were not significant (*p* > 0.05), but the sound comfort decreased with the increase in the educational background of the respondents (*p* < 0.01).

There were eight kinds of sound sources first determined in the underground commercial street of Jiefang Road, Lu’an City. The frequencies of these sources in the questionnaire were background music (13 times), conversations between customers (62 times), bargaining between customers and businesses (8 times), noise (6 times), vendors selling (11 times), footsteps (3 times), the humming of the air conditioning (22 times), and the sound of placing goods (1 time). Among them, the main controllable sound sources were the background music sound and the humming of the air conditioning. From the results of the acoustic comfort survey (Table 4), people had a high degree of acoustic comfort for the background music but a low degree of acoustic comfort for the humming of the air conditioning, which provides an improved framework for the soundscape construction of underground shopping malls.

### 3.2. Comparative Analysis of Above-Ground Space and Underground Space in Hefei City

According to the questionnaire survey, 192, 100, 245, and 200 people were “fairly satisfied” with the sound environment of the mall in shopping centers, commercial complexes, commercial streets, and shopping plazas, respectively. More people were more willing to hear the sound of music than people’s hawking sound. The overall trends of the sound evaluation of the above-ground and underground malls were relatively close. Further, it was found that music was the main component in the sound environment of shopping malls, wherein the above-ground shopping malls were better than the underground shopping malls. The satisfaction evaluation of the acoustic environment for the above-ground floor was better than that for the underground floor. The satisfaction of the acoustic environment in business space was not as good as that in non-business space.

**Figure 10:**
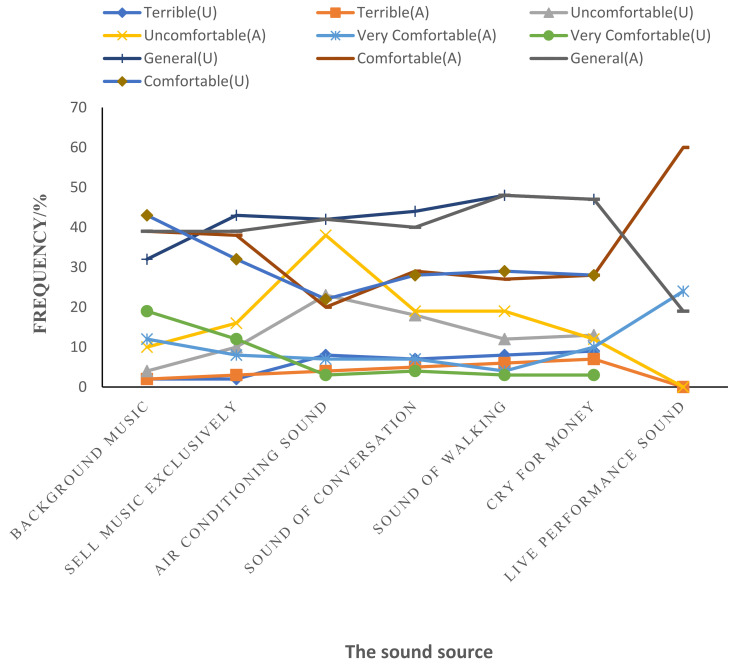
Sound source evaluation analysis diagram of shopping malls (A is above-ground shopping mall; U is for underground mall).

The results of the sound evaluation in the above-ground and underground shopping malls (Figure 10) showed that people’s level of comfort with the sound in shopping malls was generally at lower-medium levels. The evaluation of the sounds of music and air conditioning, as well as people talking, walking, and hawking, in the exclusive store became the main parts of the “general” sound perception, while the “comfortable” sounds were the sounds of background music and live performances. At the same time, the background music and the sound of live performances also accounted for the main share of customers expressing their comfort level as “very comfortable”, which reflected people’s cognitive psychology about the music sound in the mall. It can be seen that the sound associated with feeling “uncomfortable” was mainly the humming of air conditioning, as well as partly the sound of selling and talking. Regarding the evaluation of the background music sound, from “general” to “comfortable”, the above-ground and underground shopping malls, respectively, accounted for 76% of the evaluation results, but the evaluation of “comfortable” and “general” sounds in the above-ground shopping malls accounted for nearly half. In addition, the underground shopping malls were dominated by “comfortable” sounds, which was nearly 10% higher than “general” sounds, indicating that people in the underground shopping malls preferred to hear more music sounds.

**Figure 11:**
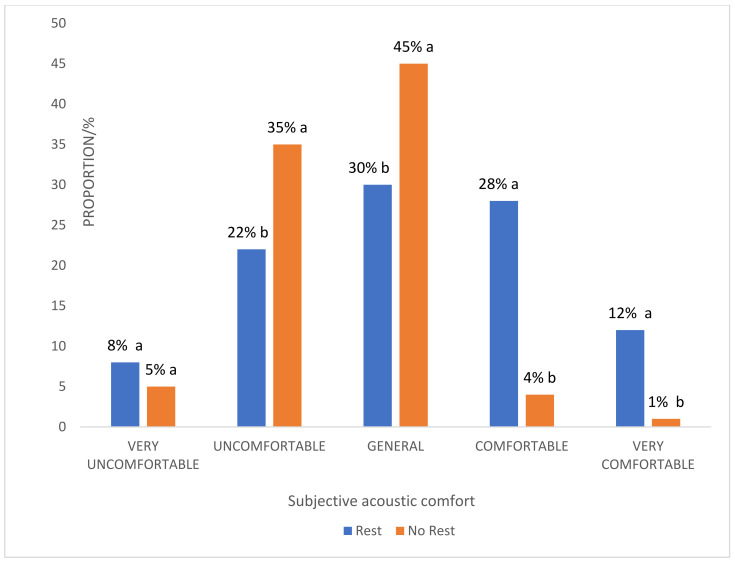
Comparison of the subjective comfort of rested and unrested customers. Different letters represent that the difference between “rest” (Group A) and “no rest” (Group B) reached significance at *p* < 0.05 level.

As shown in Figure 11, 30% of the customers who had taken a break chose “general”, 40% chose “comfortable” or “very comfortable”, and 30% chose “uncomfortable” or “very uncomfortable”. Among the customers who were going to have a rest, 45% chose “general”, 40% chose “uncomfortable” and “very uncomfortable”, and only 15% chose “comfortable” or “very comfortable”. Among the people who chose “very comfortable”, 100% were customers who were taking a break. The results indicated that people who were taking a break had higher subjective acoustic comfort ratings than those who were not taking a break (*p* < 0.01). This implied that a reasonable arrangement of spatial functions in the underground shopping mall would improve the sound comfort of consumers.

## 4. Discussion

The scale of underground commercial space is usually large, so it is easy to produce acoustic defects caused by long reverberation time. If the sound absorption performance of interior decoration materials is poor, the sound energy inside the space will be reflected multiple times without attenuation between the surrounding interfaces, which will lead to the impact of reverberation sound energy on language clarity [47,48]. In general, the measures of soundscape construction in the underground shopping mall can be considered from the following aspects:

First, the characteristics of the sound field should be altered by manipulating the spatial structure. Small spaces can be appropriately separated in combination with the display and sale forms of commodities. Many underground shopping malls are designed based on the business form of dividing sale spaces by brands [4]. Glass or lightweight partitions can be used to create several independent small spaces. This type of design can be effective in sound insulation and reverberation control to a certain extent and also changes the characteristics of the sound field determined by the spatial form.

**Figure 12:**
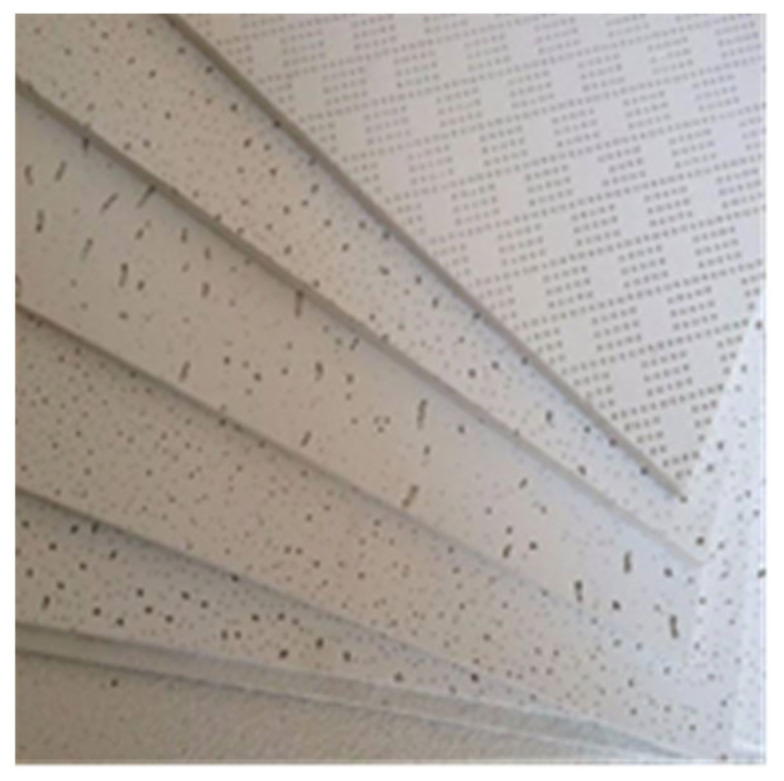
Mineral cotton fiber sound-absorbing board.

Second, sound-absorbing materials should be reasonably used in combination with indoor decoration to achieve the purpose of noise reduction. The ceiling of underground shopping malls is the focus of indoor decoration. In order to achieve a better visual effect, most suspended ceilings are made of paper-faced gypsum board, aluminum magnesium alloy, and other metal materials. From the acoustic point of view, most suspended ceilings have poor sound absorption performance in materials and structures. Considering artistry, more perforated sound absorption boards, paste mineral wool (Figure 12), and other inorganic fiber-like porous sound-absorbing materials can be used as surface materials in decorative ceilings. Materials with good sound absorption performance within the range of language sound frequency should be optimal for the purpose of sound absorption and noise reduction.

Third, in terms of commercial appliances, sound insulation, sound absorption, and noise reduction should be emphasized. In order to create a comfortable shopping environment, the ventilation and air conditioning systems of underground shopping malls are necessary, which, however, produce low-frequency equipment noise. Due to the characteristics of the sound field with slow attenuation of sound energy, underground shopping malls should adopt more rigid and comprehensive methods to control equipment noise. Sound insulation, noise elimination, and absorption measures should be strengthened from multiple perspectives such as equipment selection, machine room layout, and pipeline design to ensure low noise and high efficiency of equipment operation.

Fourth, noise problems could be solved through the combination of sound, light, and other elements in specific spaces. Appropriate acoustic design and treatment should be carried out for the atrium and other parts prone to acoustic defects. Attached underground shopping malls usually organize one or more atrium spaces in combination with escalators and sightseeing elevators, making the indoor environment more natural by introducing natural light, and reducing the sense of enclosure of the underground space by setting up vertical connecting spaces between the upper and lower floors [49,50]. These all cause potential noise interference. In the acoustic design of the atrium, acoustic defects such as acoustic focus and noise sources with high power and high decibels should be avoided. Sound-absorbing materials should be used at the enclosed surfaces of the atrium as much as possible to reduce the interference of space reverberation on language clarity.

**Figure 13:**
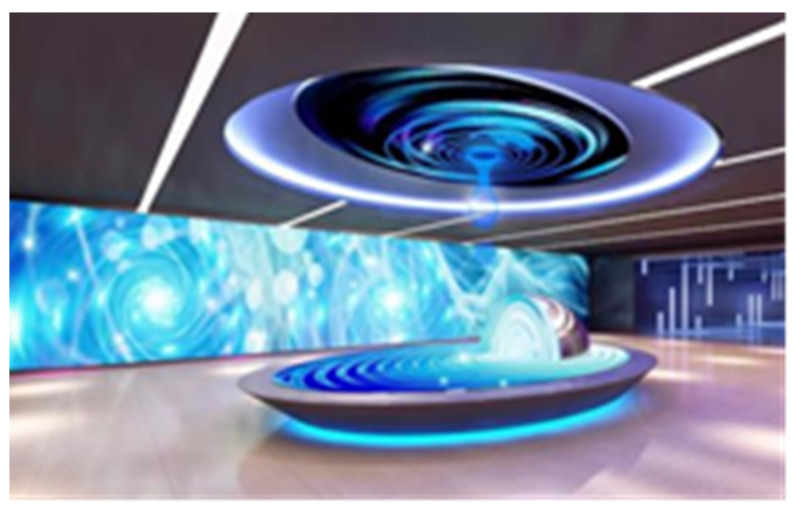
“Water Court” in Huaqiang North Metro Commercial Street, Shenzhen.

Fifth, natural sounds should be introduced to improve the soundscape quality of the underground commercial space and reduce people’s negative moods. Some voices that people do not like should be purposefully weakened, and meaningful sounds that people like should be retained. Of course, attention should be paid to ensuring that the sound pressure level in the underground space would not increase due to the increase in sound source types [51,52,53,54,55]. This will make it noisier. Different natural sounds can be introduced by taking advantage of the natural outdoor environment characteristics that people like or the landscape characteristics at different seasons. For example, the sounds of running water and birds can be used to shield some sounds that people do not like (such as the sound of air conditioning devices). To a certain extent, this can reduce the uncomfortable and anxious feeling of people in an underground, closed environment because they cannot experience nature. Accordingly, landscape elements matching the introduced natural sound should be included in the spatial design, as used, for instance, in the water atrium in Shenzhen Huaqiang North Metro Commercial Street (Figure 13). A waterscape waterfall on the wall allowed customers to experience the real natural landscape, so that sound could be heard and scenery could be followed.

Sixth, background music should be used to ensure the underground commercial space is diversified and interesting. As for the selection of such music, it is necessary to consider the type (classical music or pop music) and strength [49]. Most people do not like loud sounds from speakers [56,57,58]. The environmental sound pressure level can be set at about 65 dB. A low-frequency music sound is often not enough to suppress the noise, while a high-frequency level can make the sound of music overwhelm other environment noises [59,60]. In terms of playing, different background music can be played according to the changes in the landscape in the four seasons, so that people can have an artistic conception of touching the scene. For example, in the spring, when flowers are in full bloom, the waltz piece “The Sound of Spring” can be played, so that customers can feel the natural environment.

The seventh point worth highlighting is to integrate regional cultural resources, combine material culture with non-material culture, and then create a “soundscape space” with a cultural connotation. Human beings live in a specific urban and social environment. Urban space will inevitably bear people’s emotional and cultural needs. The introduction of cultural and artistic elements in the underground space will more easily arouse people’s resonance on the spiritual level, enhance people’s sense of identity with the space, and ultimately turn the underground commercial space into a glamorous living environment. For example, a space specifically for leisure and entertainment (e.g., resting space and stage space) can be built in underground commercial spaces, and local cultural performance activities that constitute “intangible cultural heritage” (local characteristic dramas) can be performed in a timely manner. To a certain extent, not only can this promote local cultural characteristics, but it can also give the soundscape of an underground space a certain “cultural connotation”.

## 5. Conclusions

In our study, the subjective comfort level showed an inverse U trend when the sound pressure level increased from low to high in the underground commercial street. When the ambient sound pressure level was about 65 dB, the subjective comfort level peaked. People had a high degree of acoustic comfort when listening to background music, but a low degree of comfort was associated with the humming of air conditioning, which provided an improved direction for the soundscape construction of underground shopping malls. The overall trend of the sound evaluation of the above-ground and underground malls was similar. It was found that music was the main component in the sound environment of shopping malls, and above-ground shopping malls were better than underground shopping malls. People who were taking a break had higher subjective acoustic comfort ratings than those who were not taking a break. In future research, we will continue to study how to use music to create and transform the sound scene of underground spaces.

The density of people in underground commercial space is generally high, which usually produce a lot of noise. Therefore, it is necessary to control the sound pressure level of the environmental noise to a certain level to avoid adverse health effects on people. This also enables shoppers to maintain better psychological and physiological comfort.

The construction of soundscape conforms to the general design concept, just like the Dutch architect Herman Hertzberger said, “Design is to discover what people and things want, forms naturally emerge, and there is really no need to create anything–just observe carefully” [61]. With the development of modern cities, Chinese residents are gradually shifting from the general level of demand for the number of public spaces in human settlements to a higher level of cultural edification and aesthetic enjoyment. A humanistic approach toward the construction of residents’ consumption spaces can bring them a sense of identity and belonging. At the same time, how to reasonably develop and use the urban underground commercial space and create a humane consumption space is of great significance for improving the comprehensive carrying capacity of the city, optimizing the urban environment, and building a more livable city.

## Figures and Tables

**Table 1:** Sound scale of underground commercial space Jiefang Road in Lu’an City.

Questions	Likert Scale Classification of Answers and Score
Q1. Subjective evaluation of sound clarity	Very clear (5), Clear (4), General clarity (3), Unclear (2), Very unclear (1)
Q2. Comments on the speed of background music	Fast (5), Faster (4), General velocity unclear (3), Relieve (2), Very soothing (1)
Q3. Subjective loudness evaluation of sound	Very loud (5), Loud (4), Not very loud (3), Quiet (2), Very quiet (1)
Q4. Evaluation of subjective acoustic comfort	Very comfortable (5), Comfortable (4), General comfortable (3), Uncomfortable (2), Very uncomfortable (1)

**Table 2:** Study cases and sound sources.

Instance	Place	Primary Sound Source	Layer	Number of Visitors
1	Shopping center	Background music, air conditioning sound, people talking, walking sound	The first floor underground and the third floor above ground	202
2	Commercial complex	Background music, store music, conversation, walking	The first floor underground and the third floor above ground	112
3	High street	Background music, shouting, live music, walking	The first floor underground and the first floor above ground	260
4	Shopping plaza	Store music, shouting, walking, talking	The first floor underground and the fourth floor above ground	308

**Table 3:** KMO and Cronbach’s alpha.

KMO and Bartlett’s Test			Reliability Statistics		
Kaiser–Meyer–Olkin measure of sampling adequacy		0.755	Cronbach’s alpha	Cronbach’s alpha based on standardized items	N of Items
Bartlett’s test of sphericity	Approx. chi-square	394.404	0.941	0.953	4
	df	6			
	Sig.	0.000			

**Table 4:** Acoustic comfort of background music and air conditioning noise (%).

Sound Source	Comfortable and Very Comfortable	General Comfort	Uncomfortable and Very Uncomfortable
Background music	43.7	41.5	14.8
Air conditioning sound	11.4	43.5	45.1

## Data Availability

Not applicable.
